# Predictors of lymphocyte count recovery after dimethyl fumarate-induced lymphopenia in people with multiple sclerosis

**DOI:** 10.1007/s00415-021-10412-0

**Published:** 2021-01-26

**Authors:** Matteo Lucchini, Luca Prosperini, Maria Chiara Buscarinu, Diego Centonze, Antonella Conte, Antonio Cortese, Giorgia Elia, Roberta Fantozzi, Elisabetta Ferraro, Claudio Gasperini, Antonio Ianniello, Doriana Landi, Girolama Alessandra Marfia, Viviana Nociti, Carlo Pozzilli, Marco Salvetti, Carla Tortorella, Massimiliano Mirabella

**Affiliations:** 1 Multiple Sclerosis Center, Fondazione Policlinico Universitario “A. Gemelli” IRCCS, Largo Agostino Gemelli 8, 00168 Rome, Italy; 2 Università Cattolica del Sacro Cuore, Rome, Italy; 3 S. Camillo Forlanini Hospital, Rome, Italy; 4 Center for Experimental Neurological Therapies, NESMOS, S. Andrea Hospital, Rome, Italy; 5 Department of Systems Medicine, University of Rome “Tor Vergata”, Rome, Italy; 6 IRCCS Neuromed, Pozzilli, Isernia, Italy; 7 Department of Human Neurosciences, Sapienza University, Rome, Italy; 8 San Filippo Neri Hospital, Rome, Italy; 9 Multiple Sclerosis Center, Ospedale S. Andrea, Rome, Italy; 10 Multiple Sclerosis Clinical and Research Unit, Fondazione Policlinico di Tor Vergata, Rome, Italy; 11 Department of Neurology and Psychiatry, Sapienza University, Rome, Italy

**Keywords:** Multiple sclerosis, Dimethyl fumarate, Lymphopenia, Real-world study

## Abstract

**Background:**

Dimethyl fumarate (DMF) is an oral drug approved for Relapsing Multiple Sclerosis (RMS) patients. Grade III lymphopenia is reported in 5–10% DMF-treated patients. Data on lymphocyte count (ALC) recovery after DMF withdrawal following prolonged lymphopenia are still scarce.

**Objectives:**

To characterize ALC recovery and to identify predictors of slower recovery after DMF interruption.

**Methods:**

Multicenter data from RMS patients who started DMF and developed lymphopenia during treatment were collected. In patients with grade II–III lymphopenia, ALCs were evaluated from DMF withdrawal until reaching lymphocyte counts > 800/mm^3^.

**Results:**

Among 1034 patients who started DMF, we found 198 (19.1%) patients with lymphopenia and 65 patients (6.3%) who discontinued DMF due to persistent grade II–III lymphopenia. Complete data were available for 51 patients. All patients recovered to ALC > 800 cells/mm^3^ with a median time of 3.4 months. Lower ALCs at DMF suspension (HR 0.98; *p* = 0.005), longer disease duration (HR 1.29; *p* = 0.014) and prior exposure to MS treatments (HR 0.03; *p* = 0.025) were found predictive of delayed ALC recovery.

**Conclusion:**

ALC recovery after DMF withdrawal is usually rapid, nevertheless it may require longer time in patients with lower ALC count at DMF interruption, longer disease duration and previous exposure to MS treatments, potentially leading to delayed initiation of a new therapy.

## Introduction

Several injectable, oral, and infusible Disease-Modifying Drugs (DMDs) approved for the treatment of Relapsing Multiple Sclerosis (RMS) can result in a decrease of absolute lymphocyte count (ALC) with different mechanism of action [1]. Lymphopenia is defined as a decrease in number of lymphocytes under lower limit normal (LLN) for a specific laboratory, and can be classified from grade I to IV following ALC value [2]. Clinicians need to carefully monitor ALC in patients treated with DMDs because lymphopenia can be associated with an increased risk of opportunistic infection [1].

Dimethyl fumarate (DMF) is an oral drug who demonstrated a class I evidence of efficacy for RMS patients in terms of annualized relapse rate (ARR) and radiological activity in two independent randomized clinical trials [3, 4].

In clinical trials, a reduction in ALC of approximately 30% within 1 year of treatment was reported and about 4–5% of patients reported a grade III lymphopenia [5]. DMF does not affect all lymphocyte subsets uniformly. CD8 + T cells are the most profoundly affected and CD4 +, B-lymphocyte, myeloid, and natural killer populations are also shifted toward a more anti-inflammatory state [6].

Recently, DMF-associated lymphopenia has gained clinicians’ interest due to few reports of Progressive Multifocal leukoencephalopathy (PML) in RMS patients treated with MS following prolonged lymphopenia [7–9]. Lymphopenia incidence rate in post-marketing studies is extremely variable, ranging from 10 to 30%, and even grade III lymphopenia is variable from 2.5 to 11% [10–12]. Advanced age, lower baseline ALC and female sex seem to increase the risk of DMF-induced lymphopenia [10, 13]. There was no strict indications in the management of DMF-induced lymphopenia from regulatory authorities. As for EMA prescribing information, ALC is required before starting DMF and every 3 months during treatment. DMF should be interrupted in patients with lymphocyte counts < 500/mm^3^ persisting for more than 6 months and the benefit/risk in patients with lymphocyte counts ≥ 500/mm^3^ and < 800/mm^3^ for more than 6 months should be assessed [14].

FDA prescribing information suggest obtaining ALC every 6 months after starting treatment and considering the interruption of DMF in patients with lymphocyte counts less than 0.5 × 109/L persisting for more than 6 months [15].

These statements stress the need of strict ALC monitoring but leave the clinician different possible interpretation in timing of DMF interruption. DMF prescribing information form recommends ALC monitoring during treatment and after DMF discontinuation because of lymphopenia until ALC normalization.

Only two studies investigated the features of lymphocyte count recovery after DMF-induced lymphopenia and evidence is still scarce. One study analyzed ALC data of the patients included in ENDORSE study (extension study of DMF RCTs) and the other was a monocentric study that evaluated data of 11 patients who discontinued DMF following prolonged grade III lymphopenia [16, 17].

This multicentric study aims to characterize the temporal dynamic of ALC recovery and to identify predictors of slow recovery after DMF interruption following prolonged lymphopenia.

## Methods

### Study design

This was an independent, multi-center, retrospective, post-marketing, observational study. We retrospectively analyzed data of patients affected by RMS [18] regularly attending seven tertiary MS outpatient clinics in Central Italy (Fondazione Policlinico 'A. Gemelli' IRCCS, S. Andrea Hospital, S. Camillo-Forlanini Hospital, Policlinico Umberto I, Policlinico Tor Vergata, S. Filippo Neri Hospital, Rome and IRCCS Neuromed, Pozzilli) who started DMF from October 2012 to December 2017. Clinical, laboratory and MRI data were collected by each MS center, following the local DMF monitoring plan and hospital guidelines.

### Participants

We collected data of patients with RMS who started DMF as first treatment (naïves) or were switched from other disease modifying drugs (DMDs). We excluded patients who received the first treatment prescription by one MS center but were lost at follow-up.

At baseline, we recorded the following clinical and demographical variables: sex, age, time since first symptom, EDSS score, relapses in the year before starting DMF, absence/presence of Gd + lesions, previous treatment history to classify patients in naïve or switchers.

### ALC monitoring

Lymphopenia was classified according to the common terminology criteria used for adverse events definition [2]. An ALC lower than LLN but higher than 800/mm^3^ defines a grade I lymphopenia, an ALC between 500 and 800/mm^3^ grade II lymphopenia, an ALC between 200 and 500/mm^3^ grade III and an ALC under 200/mm^3^ grade IV lymphopenia. Time interval between DMF start and first observation of lymphopenia was calculated.

ALCs longitudinal data of patients with grade II–III lymphopenia were collected from DMF withdrawal until reaching lymphocyte counts > 800/mm^3^. Mean time interval between DMF interruption and ALC normalization was calculated. We excluded patients with incomplete data (less than three ALC counts unless ALC count normalized within 60 days from DMF withdrawal) or who started a new DMD before reaching an ALC > 800/mm^3^.

### Statistical analysis

Continuous variables were described as mean ± standard deviation unless otherwise specified. Dichotomic or categorial variables were expressed as frequencies. Differences between lymphopenic and non-lymphopenic patients were explored with *t* test for independent groups and Chi-squared test as appropriate.

Cox proportional hazards model (stratified by Centre) was carried out to investigate which baseline (i.e., at treatment start) variables were associated with the development of lymphopenia during treatment with DMF. We evaluated predictors of slower ALC recovery (longer than 4 months) through a Binary Logistic Regression model.

All two-tailed *p* values < 0.05 were considered as significant, without correction for multiple comparisons considering the exploratory study design. Data were analyzed using the Statistical Package for Social Sciences, version 16.0 (IBM SPSS, Inc., Chicago, Ill., USA).

## Results

### Participants

We evaluated clinical data of 1034 MS patients. Baseline demographic and clinical characteristics of whole study population are described in Table 1. After a median follow-up of 34 months, 198 (19.1%) developed lymphopenia; 51 (4.9%), 87 (8.4%), and 60 (5.8%) patients, respectively, had grade I, II and III lymphopenia (14.2% grade II and III combined). Mean time to onset of lymphopenia was 11.3 ± 7.5 months.

**Table 1:** Whole patient cohort and lymphopenic vs non-lymphopenic patients

Patients	Whole cohort (*n* = 1034)	Normal ALC (*n* = 836)	Lymphopenia = 198	*p* value
Female sex [*n* (%)]	723 (69.9)	569 (68.1)	154 (77.8)	**0.007**
Age, years	38.8 (10.2)	38.0 (10.1)	42.1 (10.1)	** < 0.001**
Time since first symptom, years	9.5 (8.0)	9.4 (7.9)	9.9 (8.2)	0.407
EDSS score	1.94 (1.5)	1.94 (1.5)	1.92 (1.4)	0.862
Treatment naïves [*n* (%)]	318 (30.8%)	263 (31.5)	55 (27.8)	0.313
No. of relapses in previous year	0.56 (0.7)	0.54 (0.7)	0.61 (0.8)	0.218
No. of Gd + lesions	0.68 (1.3)	0.67 (1.2)	0.73 (1.4)	0.542

*EDSS *Expanded Disability Status Score,* ALC *Absolute lymphocyte count,* Gd* + gadolinium-enhancing lesion

All values are reported as mean (standard deviation) unless indicated otherwise

In bold are reported significant difference at a two-sided *α* level < 0.05

Older age (HR 1.03; 95% CIs 1.02–1.05; *p* < 0.001) and female sex (HR 1.55; 95% CIs 1.10–2.18; *p* = 0.012) were significant independent baseline predictors of lymphopenia development during DMF treatment (Table 2) through Cox regression analysis.

**Table 2:** Cox regression models (stratified by MS center) to develop lymphopenia during DMF treatment

Patients	*n* = 1134		
	HR	95% CIs	*p *value
Age (each year)	1.03	1.02–1.05	** < 0.001**
Disease duration (each year)	0.99	0.97–1.01	0.484
Female sex	1.55	1.10–2.18	**0.012**
EDSS	1.02	0.92–1.14	0.655
Naïves	1.34	0.94–1.91	0.103

*MS* multiple sclerosis, *DMF* dimethyl fumarate, *HR* hazard ratio, *95% CIs* 95% confidence intervals, *EDSS* Expanded Disability Status Score

In bold are reported significant difference at a two-sided *α* level < 0.05

### Lymphocyte count recovery

Sixty-five patients (6.3%) discontinued DMF with persistent grade II or III lymphopenia. Fourteen patients were excluded because incomplete data or loss at follow-up after DMF discontinuation.

**Fig. 1:**
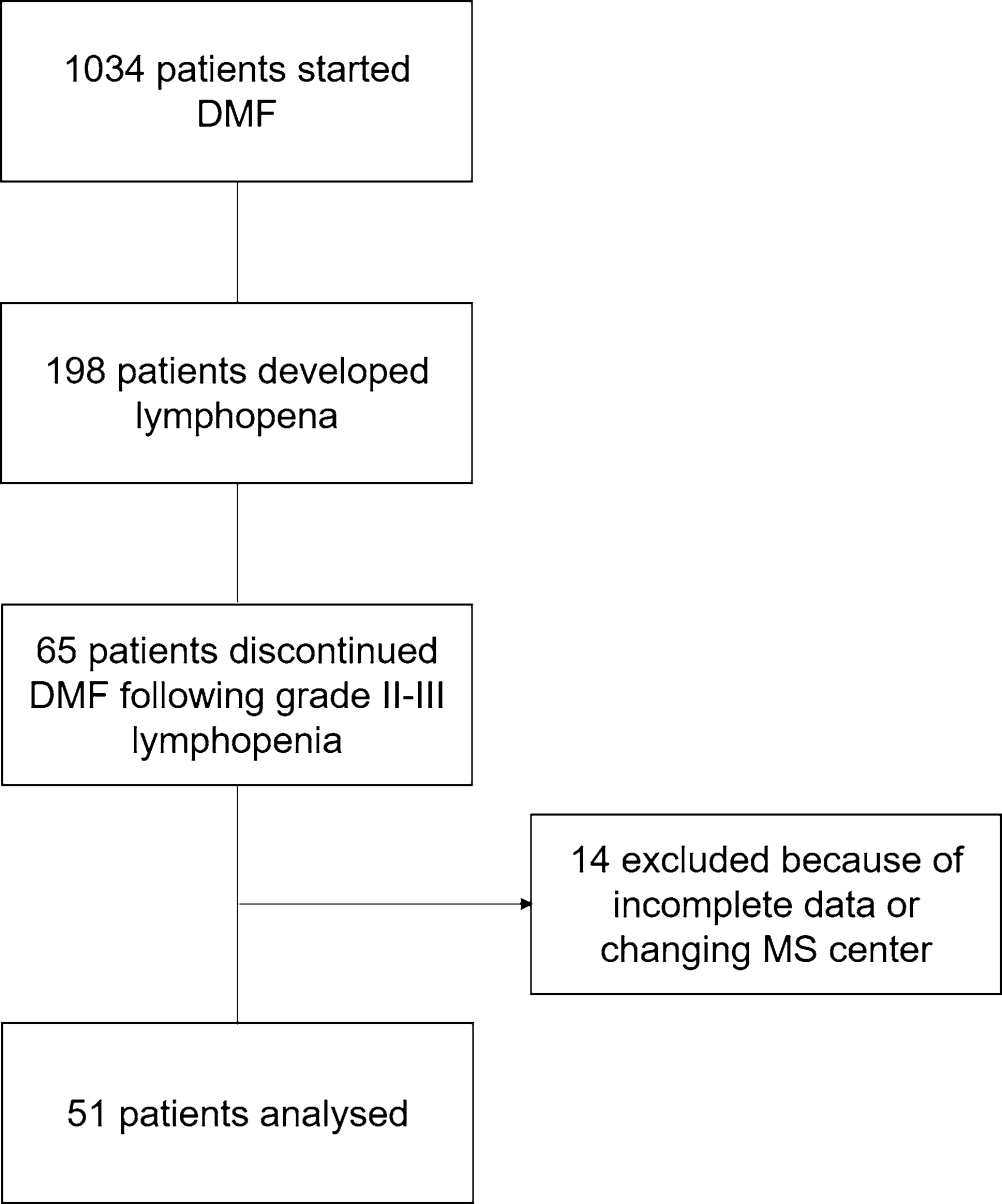
Study flow-chart

We evaluated ALCs of 51 patients from DMF withdrawal until reaching lymphocyte counts > 800/μl. (Fig. 1).

Mean age was 45.9 years with a female predominance (86.3%) and a median EDSS score of 2.0 (range 0.0–6.0). Mean disease duration was 10.9 ± 8.8 years and mean follow-up on DMF was 22.3 ± 11.9 months. Nineteen patients (37.2%) were naïve to treatment, while 32 (62.8%) were switched from another treatment; in 3 cases, the previous treatment was fingolimod and in 1 case, natalizumab (Table 3).

**Table 3:** Baseline characteristics of patients who discontinued DMF with grade II–III lymphopenia

Patients	Grade II–III lymphopenia (*n* = 51)	Grade II lymphopenia (*n* = 23)	Grade III lymphopenia (*n* = 28)
Female sex [*n* (%)]	44 (86.3)	21 (91.3)	23 (82.1)
Age, years	45.9 (10.7)	44.4 (11.6)	47.1 (10.1)
Time since first symptom, years	10.9 (8.8)	9.7 (8.4)	11.9 (9.0)
EDSS score, median [range]	2.0 [0.0–6.0]	2.0 [0.0–6.0]	2.0 [0.0–6.0]
Treatment naïves [*n* (%)]	19 (37.2)	9 (39.1)	10 (35.7)
Prior DMDs [*n* (%)] Glatiramer acetate [*n* (%)] Interferon beta 1 a [*n* (%)] Interferon beta 1 b [*n* (%)] Teriflunomide [*n* (%)] Azathioprine [*n* (%)] Fingolimod [*n* (%)] Natalizumab [*n* (%)]	9 (28.1) 13 (40.6) 3 (9.4) 2 (6.3) 1 (3.2) 3 (9.4) 1 (3.2)	5 (21.7) 4 (17.4) 1 (4.3) 2 (8.7) 1 (4.3) 1 (4.3) 0 (0.0)	4 (14.3) 9 (32.1) 2 (7.1) 0 (0.0) 0 (0.0) 2 (7.1) 1 (3.6)
Follow-up in months on DMF	22.3 (± 11.9)	23.0 (± 12.1)	21.7 (± 11.8)
Lymphopenia duration before DMF withdrawal (months)	11.2 (± 9.7)	11.2 (± 8.7)	11.2 (± 10.6)
Time to lymphocyte count recovery (months), median [range]	3.4 [0.3–13.7]	2.5 [0.3–8.4]	5.2 [0.5–13.7]

DMF dimethyl fumarate, *EDSS* Expanded Disability Status Score, *DMD* disease modifying drug

All values are reported as mean (standard deviation) unless indicated otherwise

**Fig. 2:**
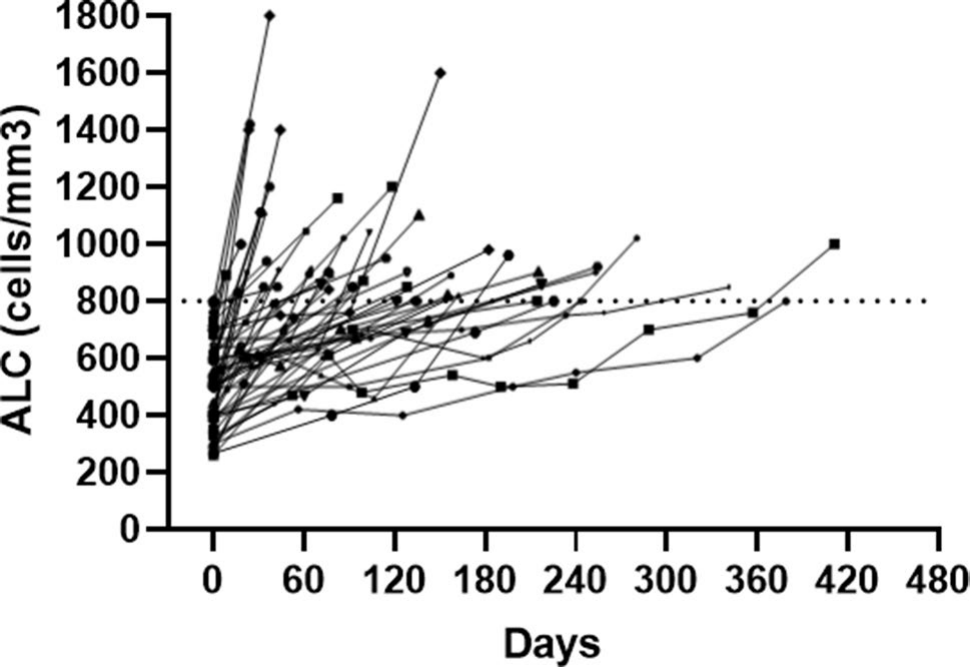
Lymphocyte count recovery after DMF withdrawal. Lymphocyte count recovery after DMF withdrawal. Each line represents the trend of lymphocyte count for a single patient. The horizontal line represents the cutoff for grade I lymphopenia (800 cells/mm^3^). *ALC* absolute lymphocyte count, *DMF* dimethyl fumarate

All patients recovered ALC to at least > 800 cells/mm^3^ during follow-up in a median time interval of 3.4 months (mean 4.2 and interquartile range 1.4–6.1) (Fig. 2).

Twenty-eight patients (55%) recovered from lymphopenia within 3 months after DMF withdrawal and 40 patients (78%) within 6 months. Two patients had a very delayed recovery lasting over a year (respectively, 12.6 and 13.7 months).

### Predictors of lymphocyte count recovery

We divided patients into two groups based on the duration of lymphopenia after DMF discontinuation and we used 4 months as cutoff to define a delayed ALC recovery. Twenty patients (39.2%) of patients have an ALC recovery longer than 4 months. Through a binary logistic regression analysis, we found that lower ALC at DMF suspension (HR 0.99; 95% CIs 0.98–0.99; *p* = 0.008), longer disease duration (HR 1.13; 95% CIs 1.01–1.27; *p* = 0.031) and prior exposure to MS treatments (HR 0.08; 95% CIs 0.01–0.54; *p* = 0.01) were significant predictors of slower ALC recovery (Table 4).

**Table 4:** Binary Logistic Regression model to evaluate predictors of lymphopenia-delayed recovery (longer than 4 months).

Patients	n=51		
	HR	95% CIs	*P*
Lymphocyte count at DMF suspension	0.99	0.98-0.99	**0.008**
Age (each year)	0.94	0.87-1.02	0.156
Disease duration (each year)	1.13	1.01-1.27	**0.031**
Female sex	0.49	0.17-1.39	0.180
EDSS	1.21	0.71-2.05	0.492
Naïves	0.08	0.01-0.54	**0.010**

*HR* hazard ratio, *95% CIs* 95% confidence intervals, *DMF* dimethyl fumarate, *EDSS* Expanded Disability Status Score

In bold are reported significant difference at a two-sided *α* level < 0.05

We cannot evaluate the impact of every single previous DMT exposure considering the low number of patients for each treatment. Notably, the two patients switching from fingolimod and developing grade III lymphopenia had both a slow recovery lasting more than 6 months (respectively, 6.1 and 7.5 months) but potential confounding interaction of age (> 50 year) and disease duration (> 10 years) cannot be excluded.

## Discussion

Several DMDs can lead to a variable degree of ALC reduction following different mechanism of action. Development of selective lymphopenia may even reflect treatment effects as part of the drug mechanism of action for several DMDs such as alemtuzumab, cladribine, ocrelizumab and fingolimod that share depleting/sequestrating mechanism of action [1].

DMF is associated with an ALC reduction within 1 year of treatment and modifies lymphocyte subset causing a higher reduction of CD19 + B cells and CD8 + T cells with an increase in CD4 + /CD8 + ratio. DMF not only alters ALC quantitatively but also qualitatively with a reduction of T and B memory cells and an expansion of T and B naïve cells [16, 19–22]

In our study, we found an incidence of 14.2% for grade II and III lymphopenia combined in over 1000 MS patients treated with DMF for nearly 3 years. As previously published by our group, we confirmed that older age and female sex are associated with higher risk of developing lymphopenia during DMF treatment [10].

Our data are consistent to RCTs and ENDORSE study as lymphopenia incidence was about 4–5% for grade III lymphopenia and near 12% for grade II and III combined [3, 4, 23].

Although there are few reports of PML in severe lymphopenic DMF-treated patients, lymphopenia was not associated with a higher risk of severe infection [7, 16, 23].

In post-marketing studies, wide variability in lymphopenia incidence rate was reported ranging from 10 to 30%, with grade III lymphopenia ranging from 2 to 10%. Different DMF exposure duration and demographic and clinical features (age at DMF initiation; previous exposure to DMDs) can explain such variability. The association between lymphopenia and patient’s age was, however, consistent in those studies [11, 12, 24, 25].

A recent paper from Sabin et al. found a 30% incidence rate for lymphopenia with 6% of grade III lymphopenia [26]. Their cohort was comparable to ours in terms of DMF exposure duration (3 years), naïve/switchers ratio (near 30% of naïve patients) and mean age (near 40 years). Our study shows a comparable rate of grade III lymphopenia while we found a lower incidence of lymphopenia including all grades. Difference among laboratories in the reference values defining lower normal lymphocyte limit may account for variability in grade I lymphopenia diagnosis [26].

While RCTs and real-world studies explored the incidence of lymphopenia during DMF treatment and the presence of potential predictors of this side effects, data regarding the restoration of ALC after DMF discontinuation for prolonged lymphopenia are still scarce.

In our study, we evaluated ALCs after DMF withdrawal of 51 patients with prolonged grade II–III lymphopenia (29 with grade III lymphopenia). Despite there are no current data regarding the level of ALC considered safe to start a new therapy, we chose a level of lymphocyte greater than 800 cells/mm^3^ to define an ALC restoration. All patients recovered to ALC > 800 cells/mm^3^ with a median time of 3.4 months. The time of ALC recovery was widely variable with eleven patients that recovered after more than 6 months, and two patients that recovered after more than a year.

Mehta et al. evaluated ALC data of patients included in ENDORSE study. They identified 207 patients with at least one ALC < 800 cells/mm^3^ (they excluded from this analysis patients with persistent grade III lymphopenia) and they found that ALC recovered to at least 800 cells/mm^3^ in 82% of patients with 63% within 4–5 weeks from DMF suspension. In this large cohort, they found 49 patients with prolonged ALC < 500 cells/mm^3^, but they have complete data only for 15 patients. In this subgroup, they found that near 40% of patients recovered to ALC > 800 within a median time of 35 weeks [16].

A monocentric study from Switzerland identified 11 patients (4.5% of their cohort) who discontinued DMF following prolonged grade III lymphopenia. Six patients were treatment naïve before starting DMF, and no one received prior treatment with cell-depleting immunotherapies (4 IFNβ-1a and 1 natalizumab). They found that ALC raised to at least 800 cells/mm3 in all patients within 6 months and with a median time of 2 months. They also identified age at withdrawal of DMF as an independent predictor for a longer duration of lymphocyte repopulation [17].

Looking at predictors of slower recovery, we found that longer disease duration and prior exposure to MS treatments were associated with longer time to ALC recovery.

### Study limits

Although our study includes clinical and laboratory data from patients attending MS tertiary outpatient clinics, we cannot rule out bias due to the retrospective study design from clinical practice. In fact, we cannot exclude a certain variability of the frequencies of ALC counts execution after DMF suspension. By excluding patients with less than three ALC counts (unless ALC count normalized within 60 days from DMF withdrawal), we have tried to minimize these potential data discrepancies.

## Conclusion

The development of lymphopenia is a critical issue not only during DMF treatment but also after its withdrawal. There is no specific wash-out period duration before starting a new DMD after DMF suspension and there are no studies who evaluated an eventual additional immunosuppressive effect of a new DMD before complete ALC restoration.

Despite ALC recovery is rapid in most cases, we highlighted that some patients, especially with longer disease duration and previous exposure to DMDs, can have a very slow recovery of lymphocyte count after DMF suspension with consequent impact in the initiation of a new DMD. In such cases, the clinician needs to carefully evaluate the risk–benefit ratio for each single patient to avoid disease activity and to reduce the potential risk (not explored) of an additional immunosuppressive effect.

Although in the majority of patients even a prolonged ALC reduction during DMF treatment may not be “per se” significantly immunosuppressive, further studies will be necessary to evaluate whether ALC recovery after lymphopenia related DMF suspension may correlate with specific previous treatments and individual patient characteristics possibly helping clinicians in better DMD sequencing.

## Data Availability

All data are available to researchers on request for the purpose of reproducing the results or replicating the procedure by directly contacting the corresponding author.
